# Dental Department of Vanderbilt University

**Published:** 1880-04

**Authors:** B. R. Freeman

**Affiliations:** Sec'y.


NASHVILLE, March 4, 1880.

The Dental Department of Vanderbilt University held its First Commencement Exercises in the Chapel of the University on Wednesday Evening, February 25th.

The Dean, D. W. H. Morgan, reported fifteen matriculates, of which number the following five were entitled to the degree of Doctor of Dental Surgery:

A. T. Kline, D. L. B. Blakemore, T. E. Gabamiss, R. B. Lees, M. D., J. H. Webber, of Tennessee.

The address on the part of the Faculty was delivered by Prof. D. R. Stubblefield, and the valedictory by D. L. B. Blakemore.

Messrs. Herman & Morrison Bros, of Tennessee Dental Depot offered two prizes, one for the best qualified second course student, and one for the first course student most proficient in all branches taught in the school. A. T. Kline received the first, an S. S. White engine, and Mr. J. P. Bailey the second a Whitney vulcanizer.

The first prize for the best gold fillings made in the Infirmary, offered by D. Henry W. Morgan, was won by D. L. B. Blakemore. L. G. Anderson, a first course student, received the second, a copy of Tyson's Celebrated Doctrine.

B. R. FREEMAN, *Sec'y.*

